# Dietary supplementation with fermented compound Chinese herbal medicine reshapes the gastrointestinal microbiota and enhances growth in suckling lambs

**DOI:** 10.1128/spectrum.03889-25

**Published:** 2026-06-30

**Authors:** Yinglian Wu, Yan Wang, Rongyan Qin, Limeng Liu, Lele Wang, Yanfeng Liu, Wenqi Wang, Qiyu Diao

**Affiliations:** 1 Feed Research Institute, Xinjiang Academy of Animal Science, Urumqi, China; 2 Xinjiang Key Laboratory of Herbivorous Livestock Feed Biotechnology, Urumqi, China; 3 Institute of Feed Research of Chinese Academy of Agricultural Sciences 12661, Beijing, China; Huazhong University of Science and Technology, Wuhan, China

**Keywords:** fermented compound Chinese herbal medicine, lambs, growth performance, antioxidant capacity, gastrointestinal microbiota

## Abstract

This study investigated the effects of a fermented compound Chinese herbal medicine (FCHM) on growth performance, antioxidant capacity, immune function, and gastrointestinal microbiota in suckling lambs. FCHM consisted of 10 herbs fermented with *Candida utilis* and *Bacillus subtilis*. Sixty twin Hu lambs (15 days) were randomly fed a basal diet (CON) or the diet supplemented with 0.6% FCHM (Treat) for 45 days. The results indicated that the Treat group exhibited a significant increase in average daily gain (ADG) (*P* < 0.05). Serum analyses revealed elevated levels of growth hormone (GH), insulin-like growth factor-1 (IGF-1), total antioxidant capacity (T-AOC), superoxide dismutase (SOD), glutathione peroxidase (GSH-Px), and glucose (GLU), whereas malondialdehyde (MDA) and pro-inflammatory cytokines (IL-6 and TNF-α) were reduced (*P* < 0.05). In the duodenal mucosa, SOD and GSH-Px activities and T-AOC levels were significantly elevated, while MDA content was notably decreased (*P* < 0.05). Ruminal fermentation profiles showed increased concentrations of propionate and total volatile fatty acids (TVFA) in the Treat group (*P* < 0.05). Microbiome analysis revealed that FCHM supplementation selectively modulated the ruminal microbial community, enriching beneficial genera such as *Prevotellaceae_UC*G-003 and Butyrivibrio, while reducing the abundance of potentially harmful genera like Streptococcus, despite no significant changes in the overall community diversity. Metagenomic sequencing further demonstrated the enrichment of KEGG enzymes and carbohydrate-active enzyme genes involved in carbohydrate metabolism and propionate biosynthesis. Correlation network analyses revealed significant associations among specific microbial taxa, serum antioxidant, immune biomarkers, and growth performance. In conclusion, dietary FCHM supplementation improves growth performance in suckling lambs by optimizing ruminal fermentation patterns, selectively regulating gastrointestinal microbiota, and enhancing systemic antioxidant capacity. These findings support the potential of FCHM as a functional feed additive in lamb production systems.

**IMPORTANCE:**

Enhancing growth performance and ensuring gastrointestinal health during the suckling period are critical for lamb productivity and welfare. In the context of the antibiotic-free mandate in animal feed, we evaluated the effects of a fermented compound Chinese herbal medicine (FCHM) on growth, antioxidant status, immune parameters, and gastrointestinal microbiota in lambs. Our findings demonstrate that FCHM improves average daily gain, enhances systemic and mucosal antioxidant capacity, and modulates ruminal and hindgut microbiota by enriching beneficial taxa and suppressing potentially harmful bacteria. These effects are linked to upregulated microbial functions in carbohydrate metabolism and propionate biosynthesis. This study provides a microbial-based mechanism for FCHM as a natural feed additive to promote lamb growth and gastrointestinal resilience, offering a sustainable strategy to support early-life development in ruminant production systems.

## INTRODUCTION

The suckling phase is a critical period for lamb growth and development. During this stage, lambs are highly susceptible to diarrhea, which consequently compromises gastrointestinal development, reduces the apparent digestibility of dietary nutrients, induces gut microbiota dysbiosis, and triggers systemic oxidative stress, ultimately impairing animal health and productivity (1). In recent years, Chinese herbal medicines have garnered significant research interest. Notably, Traditional Chinese Herbal Medicines (TCHMs) are abundant in active compounds, including polysaccharides, flavonoids, and trace elements, which contribute to their immunomodulatory, anti-inflammatory, and antioxidant properties (2, 3). These bioactivities thereby lay a solid foundation for developing herbal feed additives aimed at promoting growth and enhancing immune function in livestock.

Moreover, the fermentation of active herbal ingredients by microorganisms serves to enhance the bioactivity of the resulting products. This process not only effectively mitigates the toxic side effects associated with certain herbs but also facilitates the degradation of plant cell walls, thereby promoting the release of bioactive natural compounds (4–6). In the context of China’s prohibition on antibiotic use in animal feed since 2020, Chinese herbal medicines have correspondingly been demonstrated to exert beneficial effects on ruminant health as feed additives, given their safety profile and capabilities in growth promotion and antioxidant capacity enhancement. For instance, studies have demonstrated that supplementing fermented Chinese herbal medicine (a compound of 18 herbs including *Astragalus membranaceus*, *Platycladus orientalis leaves*, *Crataegus pinnatifida*, *Ligusticum chuanxiong*, and *Glycyrrhiza uralensis*) under heat stress conditions improves blood biochemical parameters and antioxidant capacity in dairy cows, consequently enhancing milk production performance (7). Separately, Luo et al. reported that fermented Chinese herb residues significantly increased serum total antioxidant capacity (T-AOC), glutathione peroxidase (GSH-Px), and superoxide dismutase (SOD) activities in Hu sheep (8). Furthermore, studies have indicated that dietary supplementation with 1.0% or 1.5% fermented Chinese herbal medicine enhances growth performance in fattening lambs under heat stress by strengthening the ruminal mucosal barrier (9). Nevertheless, despite some successful applications in ruminant nutrition, the widespread adoption of fermented Chinese herbs as feed additives remains limited in animal production systems. This constraint stems primarily from the extensive variety of herbal species, their complex phytochemical profiles, and the insufficient elucidation of their underlying mechanisms of action.

Therefore, this study formulated a compound Chinese herbal medicine comprising 10 medicinal plants, including *A. membranaceus*, *Citri Reticulatae Pericarpium, Citri Reticulatae Pericarpium Viride*, and *Atractylodis Macrocephalae Rhizoma*, which were selected for their documented benefits in promoting gastrointestinal health and enhancing immune function. Following microbial fermentation, the preparation was administered to newborn lambs. We hypothesized that enhancing antioxidant capacity through dietary FCHM is a pivotal mechanism that modulates the gastrointestinal microbiota to improve growth performance in suckling lambs. This study was conducted to elucidate these underlying processes and provide a scientific basis for the use of FCHM in neonatal lamb rearing systems.

## MATERIALS AND METHODS

### Experimental design and animal management protocols

A mixture of Chinese herbal medicine, including *A. membranaceus*, *Citri Reticulatae Pericarpium*, *Citri Reticulatae Pericarpium Viride*, *Hordeum vulgare*, *A. macrocephala*, *Massa Fermentata*, and *Berberis julianae*, was sourced from local markets and uniformly blended in a fixed ratio of 2:2:2:3:1:2:3. The microbial inoculants *Candida utilis* (≥5 × 10⁸ CFU/g, strain ATCC 22023) and *Bacillus subtilis* (≥2 × 10¹°CFU/g, strain ATCC 6633) obtained from Gaotang Huanong Bioengineering Co., Ltd., were mixed at a 1:1 ratio. The herbal mixture was ground using a 40-mesh sieve and combined according to the specified proportions, and then adjusted to a substrate moisture content of 68%. Following the addition of 1% microbial inoculant, solid-state fermentation was carried out at ambient temperature for 7 days. The fermented product was subsequently dried at 65°C for 48 h and ground into a fine powder. For the animal trial, 60 15-day twin-born Hu lambs (average birth weight: 3.25 ± 0.5 kg) with good body condition and born to second-parity ewes were selected. These lambs were randomly allocated into two treatment groups, each comprising three replicates of 10 lambs (equally divided by sex). Based on previous experimental findings from our research team indicating that a 0.6% inclusion level of the Chinese herbal additive yielded optimal results in lamb diets, this dosage was selected for the present study. Accordingly, two experimental groups were established: a control group (CON) receiving only starter feed, and a treatment group (Treat) receiving starter feed supplemented with 0.6% of the fermented herbal additive. The starter feed was formulated to meet the Nutrient Requirements of Meat Sheep (NY/T 816-2021), with detailed composition and nutrient content provided in Table 1.

**TABLE 1:** Composition and nutritional level of lamb starter feed (DM basis)

Ingredients	% Content	Nutrient levels^*b*^	% Content
Corn	41.42	CP	16.20
Soybean meal	19.00	EE	4.16
Sativa	15.00	Ash	11.29
Premix^*a*^	5.00	NDF	22.26
Yeast powder	4.00	ADF	8.93
Whey powder	3.80	Ca	1.07
Cottonseed protein	3.00	P	0.51
Glucose	2.90	GE/ (MJ/kg）	15.64
Soybean oil	2.00		
CaHPO_4_	1.00		
NaHCO_3_	1.00		
NaCl	1.00		
Lysine	0.45		
Methionine	0.20		
Mold remover	0.10		
Multivitamin	0.10		
Cysteine	0.03		
Total	100.00		

^*a*^ Premix provided with the following per kg of the diet: VA 20,000 IU, VD3 10,000 IU, VE 260 mg, C6H5NO2 250 mg, Cu 180 mg, Fe 2,000 mg, Zn 1,200 mg, Mn 1,000 mg, I 10 mg, Se 7 mg, Co 6 mg, Ca 9 mg, P 1.3 mg, NaCl 5 mg.

^*b*^ Nutritional levels are all measured values.

The starter feed and fermented herbal additive were mixed in the designated proportions to produce total mixed ration (TMR) pellets. The feeding trial lasted 45 days. Prior to its initiation, all experimental pens and surrounding facilities were thoroughly sanitized. During the trial, ewes and lambs were housed in separate pens, allowing lambs to suckle freely from their dams. Structured commingling sessions between lambs and ewes were scheduled as follows: three sessions between 15 and 22 days, two sessions between 23 and 44 days, and one session between 45 and 60 days post-birth, with each session lasting 1 h. Lambs were provided with starter feed twice daily at 09:00 and 18:00 and had *ad libitum* access to both feed and water throughout the study.

### Bioactive compounds analysis

Untargeted metabolomic profiling was conducted using an ExionLC Ultra Performance Liquid Chromatography System (SCIEX, Framingham, MA, USA) equipped with a Waters ACQUITY UPLC HSS T3 analytical column (1.8 μm, 2.1 × 100 mm). Chromatographic separation was achieved through a gradient elution method. Mass spectrometric detection was carried out using a SCIEX TripleTOF 6600 system. Data acquisition and quantitative analysis were performed using Analyst TF Software (version 1.6.3).

### Sample collection and quantification

On the first and final day of the trial (09:00), suckling lambs from all treatment groups were weighed after a 12-h fast to determine individual body weight (BW) for calculating average daily gain (ADG). Daily feed intake per replicate was recorded to estimate the average starter feed consumption per lamb. On day 45, two lambs per replicate (*n* = 12 total) were randomly selected for blood collection. Following a 12-h fast, 5 mL jugular venous blood samples were obtained and allowed to clot at room temperature for 30 min before centrifugation at 3,000 × *g* for 15 min. The resulting serum was aliquoted into 1.5 mL microcentrifuge tubes and stored at −20°C. Additionally, six lambs per group (two per replicate) were randomly selected for slaughter after a 12-h fasting period. Following exsanguination via jugular venipuncture, duodenal segments were excised, thoroughly cleared of digesta, rinsed with physiological saline, and collected for antioxidant parameter analysis. Concurrently, ruminal fluid and rectal fecal samples were obtained. Rumen pH was immediately measured using a calibrated pH meter (Model PHS-3C; Shanghai Yueping Scientific Instruments, Shanghai, China). All samples were aliquoted into sterile 1.5 mL microcentrifuge tubes and stored at −80°C for subsequent microbial DNA extraction and volatile fatty acid (VFA) analysis.

VFA concentrations were quantified by gas chromatography (Agilent 6890N, USA), while rumen ammonia-nitrogen (NH₃-N) was measured spectrophotometrically (Thermo Multiskan FC, USA). Serum GH, INS, and IGF-1 levels were determined using species-specific ELISA kits (Solarbio, China). Serum AST, ALT, CK, and GLU were analyzed with automatic biochemical analyzers (Mindray BS-420 and Perlove D-320, China). Antioxidant parameters (MDA, T-AOC, GSH-Px, and SOD) were assessed using a semi-automatic analyzer (PUS-2018, China) with kits from Nanjing Jiancheng. Serum cytokines (IL-6, IL-4, and TNF-α) and immunoglobulins (IgG, IgM, and IgA) were measured by ELISA (Tecan Infinite 200Pro, Switzerland) with kits from Suzhou Geruisi.

### DNA extraction, 16S rRNA gene amplification, and sequencing

Samples were submitted to PersonalBio Technologies Co., Ltd. (Shanghai, China) for specialized testing and bioinformatics analysis. Nucleic acids were extracted from pre-processed samples using the E.Z.N.A. Soil DNA Kit (Omega Bio-Tek, Norcross, GA, USA). The quality of extracted DNA was assessed by 0.8% (wt/vol) agarose gel electrophoresis to evaluate fragment size distribution, while DNA concentration was measured using a NanoDrop NC-2000 spectrophotometer (Thermo Fisher Scientific, Waltham, MA, USA). PCR amplification targeted the hypervariable V3–V4 region of the 16S rRNA gene using degenerate primers 338F (5′-ACTCCTACGGGAGGCAGCAG-3′) and 806R (5′-GGACTACHVGGGTWTCTAAT-3′). Amplicons were quantified via the Quant-iT PicoGreen dsDNA Assay Kit on a Microplate Reader (BioTek Synergy H1, Winooski, VT, USA), followed by library construction employing the Illumina TruSeq Nano DNA LT Library Prep Kit (San Diego, CA, USA). Bioinformatic processing of microbiome data was conducted using QIIME 2 (version 2019.4). Raw sequence data underwent stringent quality control steps, including quality filtering, denoising, merging of paired-end reads, and chimera removal. High-quality sequences were subsequently clustered into amplicon sequence variants (ASVs) with 100% sequence similarity. These ASVs were taxonomically classified and used for microbial community abundance profiling and comparative analyses.

### Metagenome sequencing

Rumen fluid samples were submitted to BGI Genomics Co., Ltd. (Shenzhen, China) for metagenomic sequencing. Genomic DNA was extracted using the E.Z.N.A. Soil DNA Kit (Omega Bio-Tek, Norcross, GA, USA). The extracted DNA was mechanically sheared to approximately 400 bp fragments using a Covaris M220 ultrasonicator (Covaris, Woburn, MA, USA). Paired-end (PE) libraries were prepared using the NEXTFLEX Rapid DNA-Seq Kit (Bioo Scientific Corporation, Austin, TX, USA). Paired-end (PE) libraries were constructed with the NEXTFLEX Rapid DNA-Seq Kit (Bioo Scientific Corporation, Austin, TX, USA). Pooled libraries were sequenced on the Illumina HiSeq X Ten platform (2 × 150 bp paired-end mode). Raw paired-end reads underwent quality control preprocessing using Fastp (version 0.23.1; https://github.com/OpenGene/fastp), including removal of low-quality bases and adapter sequences with consequent generation of high-fidelity clean data (10). Filtered reads were assembled *de novo* using MEGAHIT (v1.1.2; https://github.com/voutcn/megahit) (11), and contigs shorter than 200 bp were excluded. Open reading frames (ORFs) were predicted using MetaGeneMark (http://exon.gatech.edu/GeneMark**/**) (12). All predicted genes were clustered using CD-HIT (http://www.bioinformatics.org/cd-hit/) (13) to generate non-redundant gene sets. These sequences were then aligned against the GenBank non-redundant protein database using DIAMOND (https://github.com/bbuchfink/diamond). Functional annotation, including the identification of carbohydrate-active enzymes (CAZymes), was performed by comparative analysis with the CAZy database (http://www.cazy.org/).

### Statistical analysis

Statistical analyses of ADG, feed intake, hematological parameters, rumen fermentation characteristics, and bacterial taxonomic and functional profiles were performed using SAS software (version 9.4). Processing of 16S rRNA sequencing data was carried out on the GenesCloud platform (https://www.genescloud.cn/), while metagenomic data were analyzed using the BGI BioSYSTEMS platform (https://biosys.bgi.com/). Alpha diversity indices were calculated using QIIME2 (v2023.5; https://qiime2.org), and inter-group differences were assessed using the Wilcoxon rank-sum test. Beta diversity was evaluated using principal coordinate analysis (PCoA) based on weighted and unweighted UniFrac distance metrics, with analyses conducted through integrated pipelines in R (v4.3.1) and QIIME2 (v2023.5). To statistically assess beta diversity differences between groups, permutational multivariate analysis of variance (PERMANOVA) was performed using the adonis2 function in the R package vegan (v2.6-4) with 999 permutations based on weighted UniFrac distances, and the effect size (R²) and corresponding *P*-values were reported. Differential microbial features between treatment groups were identified using the Linear Discriminant Analysis Effect Size (LEfSe) method, applying a logarithmic LDA score threshold of >2.0 and a significance level of α = 0.05 (Kruskal–Wallis test followed by pairwise Wilcoxon rank-sum tests with Benjamini–Hochberg correction). Microbial co-occurrence networks were constructed based on statistically significant Spearman correlations (|r| > 0.5, *P* < 0.05) and visualized using Gephi software (v0.10; The Gephi Consortium, Paris, France).

## RESULTS

### Principal bioactive constituent profile

**Fig 1:**
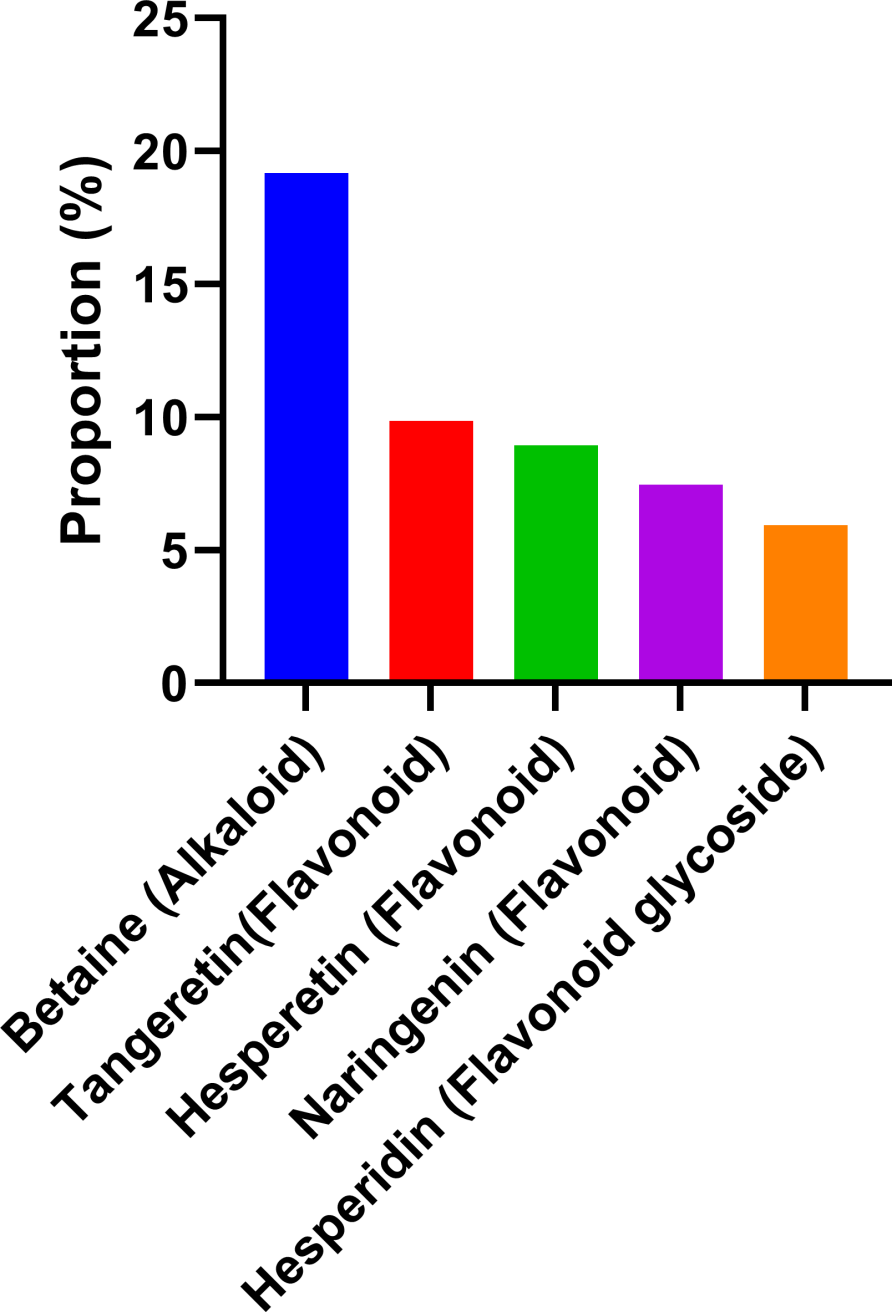
Predominant bioactive constituents (Top 5) in the fermented botanical formulation.

LC-MS analysis identified a total of 605 phytochemically active compounds in the fermented Chinese herbal compound, which were classified into 44 distinct categories. Among these, the major groups included 121 flavonoids and flavonoid derivatives, 87 alkaloids, 55 terpenoids, 46 phenolic acids, 20 amino acids and their derivatives, and 15 aromatic compounds. The five most abundant constituents, betaine, tangeretin, hesperetin, naringenin, and hesperidin, were primarily classified as alkaloids and flavonoids (Fig. 1).

### Growth performance

**Fig 2:**
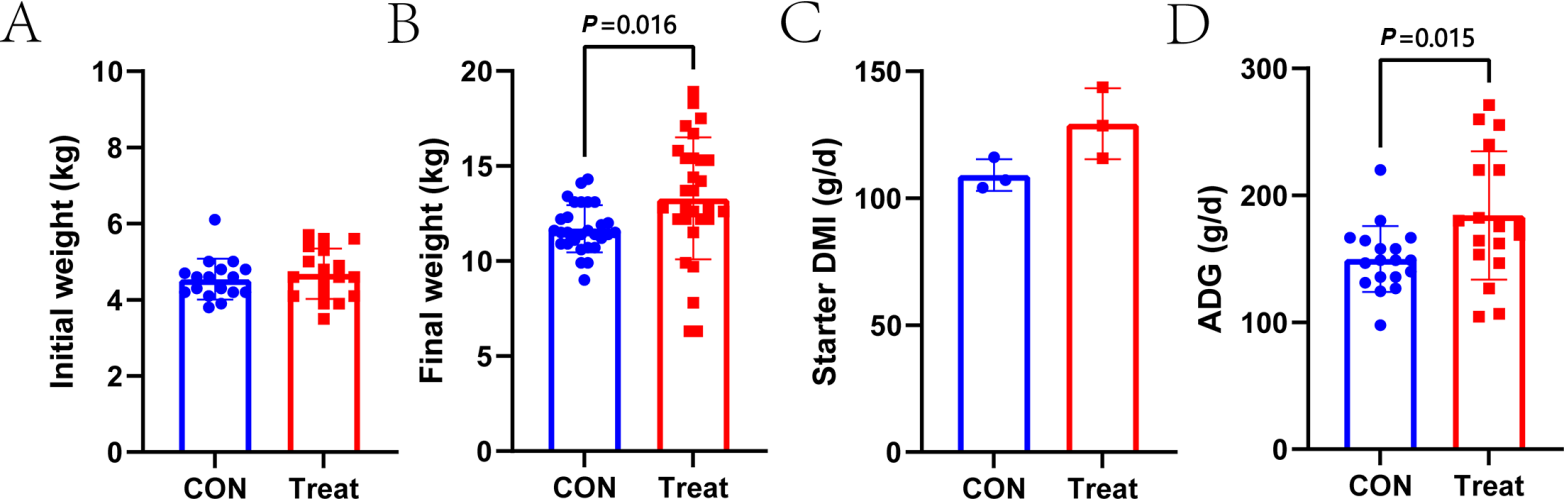
Effects of supplemental fermented Chinese herbal medicine on the growth performance of lambs. Data are presented as mean ± SEM. Group comparisons were analyzed using an independent samples *t*-test. Bar graphs comparing growth metrics in lambs with fermented Chinese herbal medicine supplementation. (**A**) Initial weight; (**B**) final weight; (**C**) starter DMI; (**D**) ADG. Treatment group shows significantly higher final weight (*P* = 0.016), dry matter intake, and average daily gain (*P* = 0.015) despite similar initial weights.

Growth performance data revealed that the Treat group significantly enhanced final body weight (t₃₄ = 2.539 , *P* = 0.016) and ADG (t₃₄ = 2.563, *P* = 0.015) in lambs compared to the CON group, while no significant differences were observed in the initial body weight or dry matter intake of the starter diet (*P* > 0.05) (Fig. 2).

### Serum biochemical and growth hormone parameters

**Fig 3:**
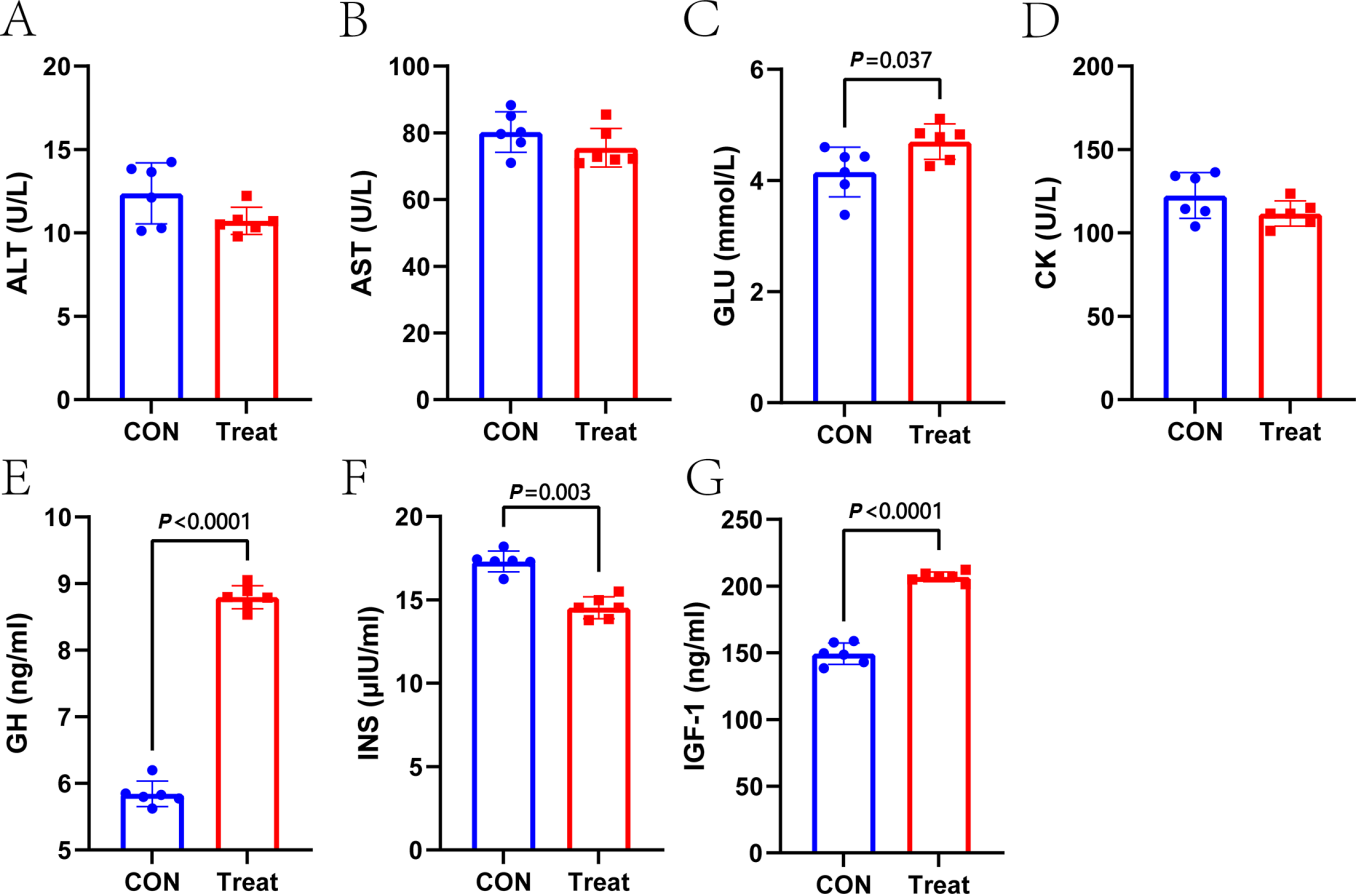
Effects of supplemental fermented Chinese herbal medicine on serum biochemical and growth hormone parameters in lambs. Data are presented as mean ± SEM. Group comparisons were analyzed using an independent samples *t*-test. Bar graphs comparing serum biochemical parameters between control and fermented Chinese herbal medicine groups in lambs. (**A**) ALT; (**B**) AST; (**C**) GLU; (**D**) CK; (**E**) GH; (**F**) INS; (**G**) IGF-1. Treatment significantly increased glucose, growth hormone, and IGF-1 levels while decreasing insulin compared to control.

As presented in Fig. 3, serum concentrations of GLU (t_10_ = 2.443, *P* = 0.035), GH (t_10_ = 28.20, *P* < 0.0001), and IGF-1 (t_10_ = 16.00, *P* < 0.0001) in the Treat group were significantly elevated, whereas the INS (t_10_ = 3.85, *P* = 0.003) level was significantly reduced compared to the CON group. In contrast, no significant differences were observed in the serum levels of AST, ALT, and CK between the groups (*P* > 0.05).

### Serum immune parameters

**Fig 4:**
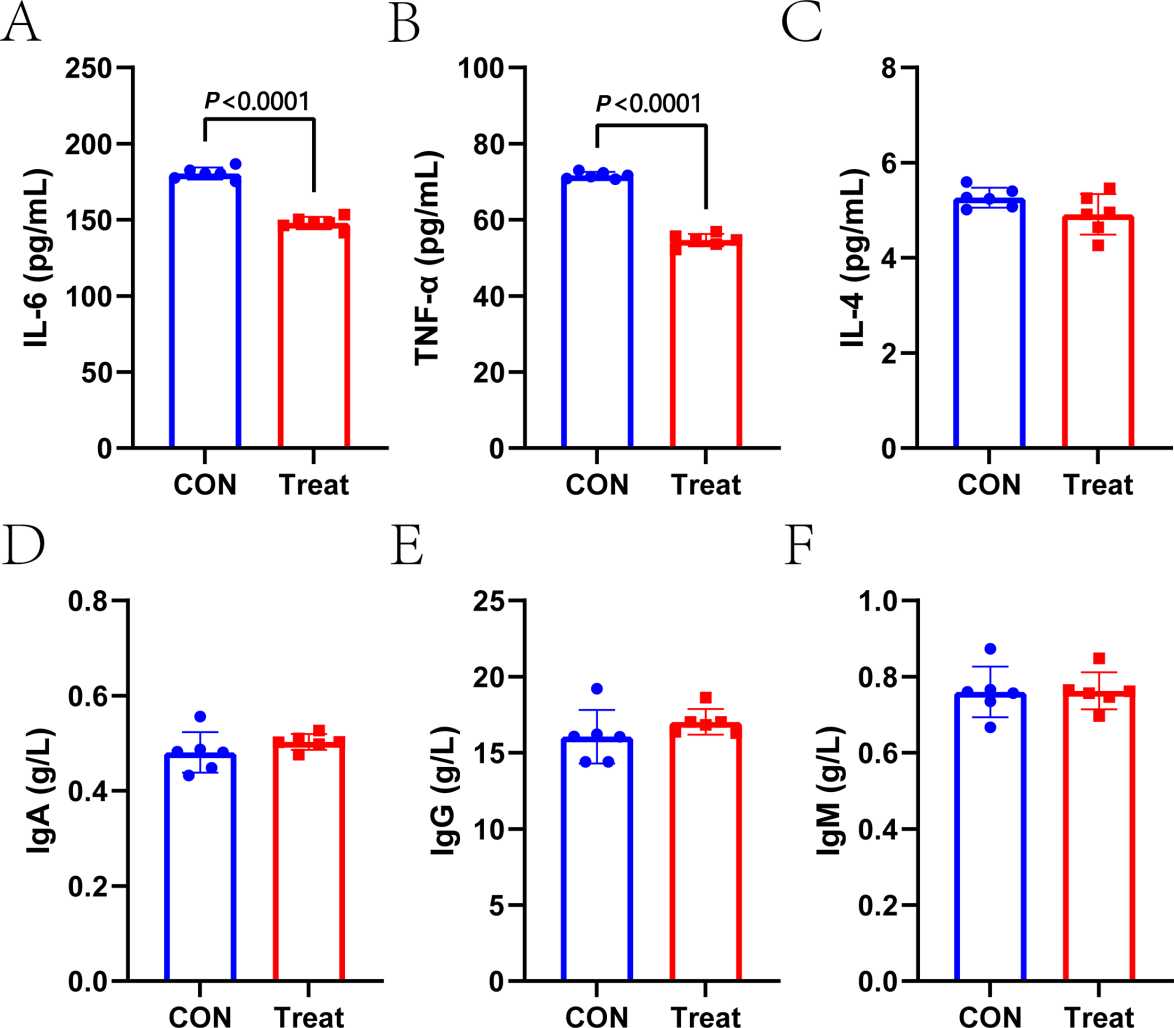
Effects of supplemental fermented Chinese herbal medicine on serum immune parameters in lambs. Data are presented as mean ± SEM. Group comparisons were analyzed using an independent samples *t*-test. Bar graphs showing fermented Chinese herbal medicine effects on lamb serum immune parameters. (**A**) IL-6; (**B**) TNF-α; (**C**) IL-4; (**D**) IgA; (**E**) IgG; (**F**) IgM. Treatment significantly reduced inflammatory cytokines IL-6 and TNF-α, while IL-4 and immunoglobulins IgA, IgG, and IgM remained unchanged.

The serum immunity results (Fig. 4) revealed that the Treat group exhibited significantly lower serum concentrations of IL-6 (t_10_ = 14.15, *P* < 0.0001) and TNF-α (t_10_ = 22.14, *P* < 0.0001) compared to the CON group. In contrast, the levels of IgA, IgG, and IgM remained comparable between the two groups (*P* > 0.05).

### Serum antioxidant parameters

**Fig 5:**
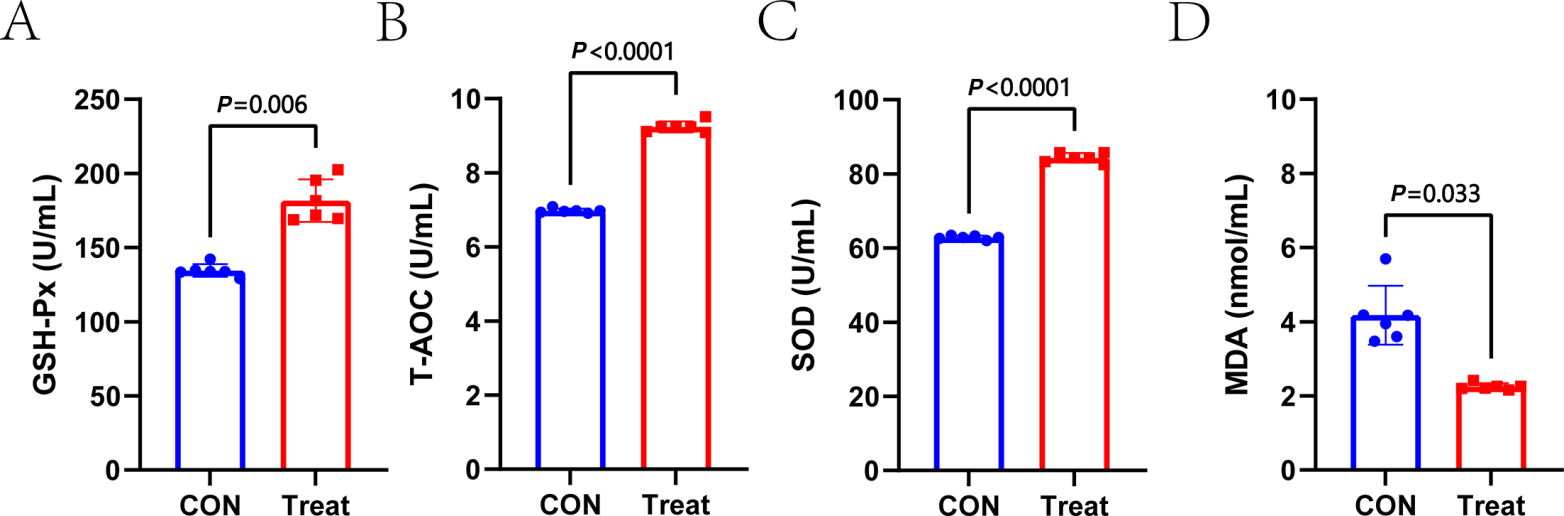
Effects of supplemental fermented Chinese herbal medicine on serum antioxidant parameters in lamb. Data are presented as mean ± SEM. Group comparisons were analyzed using an independent samples *t*-test. Bar graphs showing serum antioxidant parameters in lambs with and without fermented Chinese herbal medicine. (**A**) GSH-Px; (**B**) T-AOC; (**C**) SOD; (**D**) MDA. Treatment significantly increased GSH-Px, T-AOC, and SOD levels while decreasing MDA levels, all with statistical significance.

Serum antioxidant analysis revealed that the Treat group displayed pronounced elevations in the activities of GSH-Px (t_10_ = 3.581, *P* = 0.006), T-AOC (t_10_ = 34.32, *P* < 0.0001), and SOD (t_10_ = 37.96, *P* < 0.0001), while the MDA (t_10_ = 2.475, *P* = 0.033) level was concomitantly significantly lowered compared to the control group (Fig. 5).

### Duodenal mucosal antioxidant parameters

**Fig 6:**
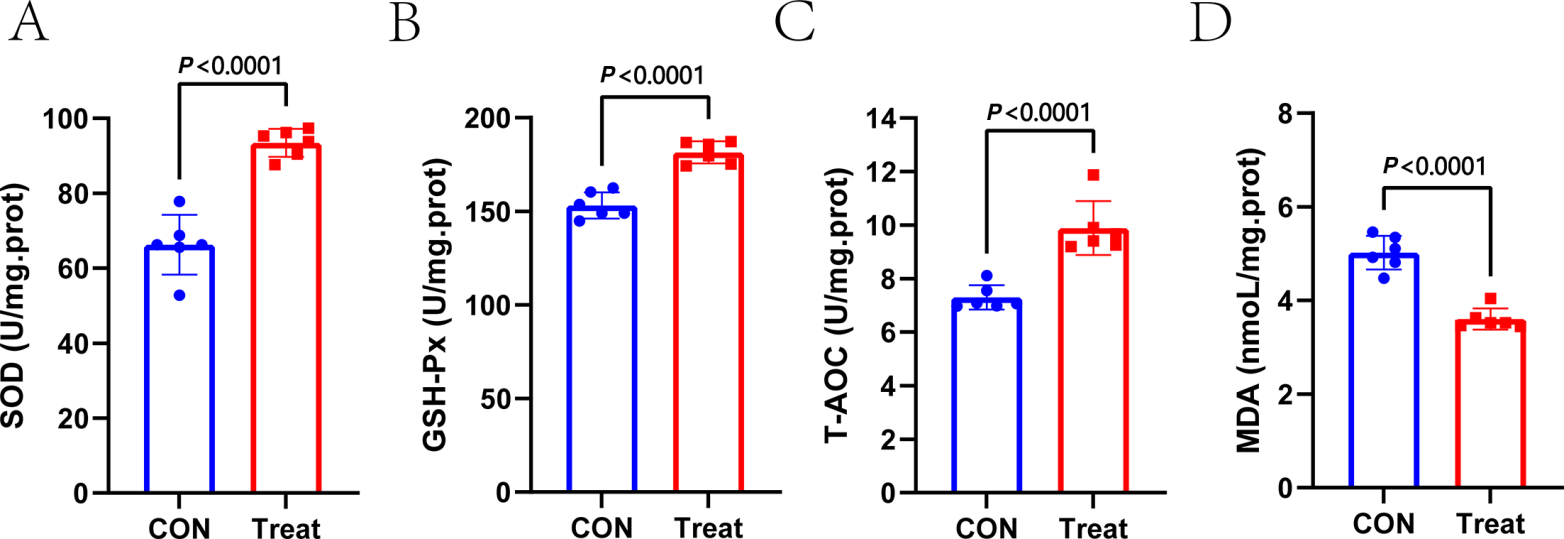
Effects of supplemental fermented Chinese herbal medicine on duodenal mucosal antioxidant parameters in lambs. Data are presented as mean ± SEM. Group comparisons were analyzed using an independent samples *t*-test. Bar graphs comparing duodenal antioxidant parameters between control and treated lambs. (**A**) SOD; (**B**) GSH-Px; (**C**) T-AOC; (**D**) MDA. Fermented Chinese herbal medicine significantly increased SOD, GSH-Px, and T-AOC levels and decreased MDA levels.

As shown in Fig. 6, compared with the control group, the Treat group exhibited significantly elevated levels of SOD (t_10_ = 7.540, *P* < 0.0001), GSH-Px (t_10_ = 7.614, *P* < 0.0001), and T-AOC (t_10_ = 5.741, *P* < 0.0001), alongside a marked reduction in MDA (t_10_ = 8.136, *P* < 0.0001) concentration in the duodenal mucosa of lambs.

### Ruminal fermentation parameters

**Fig 7:**
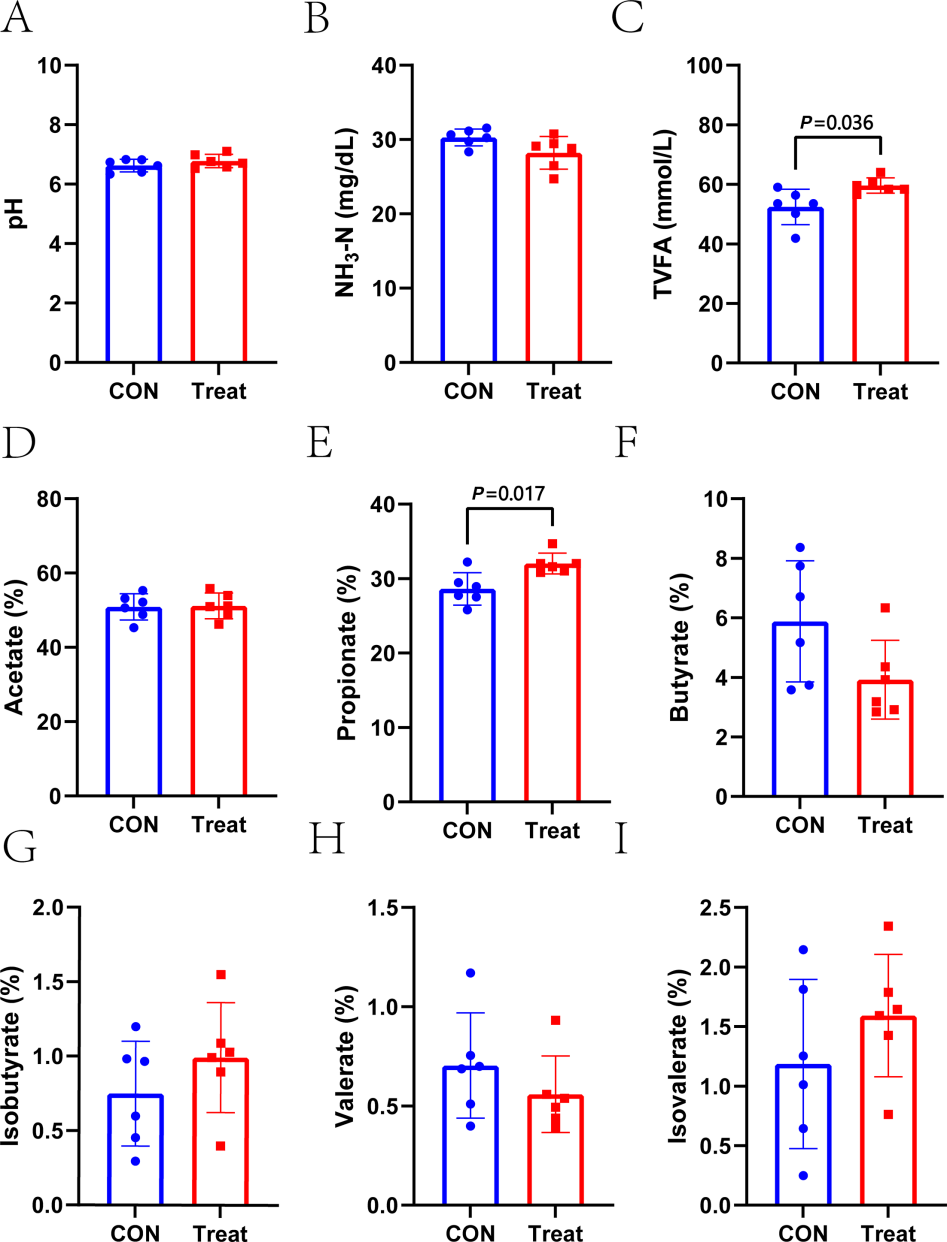
Effects of supplemental fermented Chinese herbal medicine on rumen fermentation parameters in lambs. Data are presented as mean ± SEM. Group comparisons were analyzed using an independent samples *t*-test. Nine bar graphs comparing rumen fermentation parameters between control and treated lambs. (**A**) pH; (**B**) NH₃-N; (**C**) TVFA; (**D**) Acetate; (**E**) Propionate; (**F**) Butyrate; (**G**) Isobutyrate; (**H**) Valerate; (**I**) Isovalerate. Fermented Chinese herbal medicine significantly increased TVFA (*P* = 0.036) and propionate percentage (*P* = 0.017). Other parameters showed no significant differences.

As shown in Fig. 7, the treatment group exhibited significantly higher concentrations of ruminal TVFA (t_10_ = 2.520, *P* = 0.036) and propionate (t_10_ = 2.984, *P* = 0.017) compared to the control group. However, no significant differences were observed between the groups for acetate, butyrate, isobutyrate, valerate, isovalerate, rumen pH, or NH₃-N levels (*P* > 0.05).

### Ruminal microbiota composition, diversity, and differential abundance

**Fig 8:**
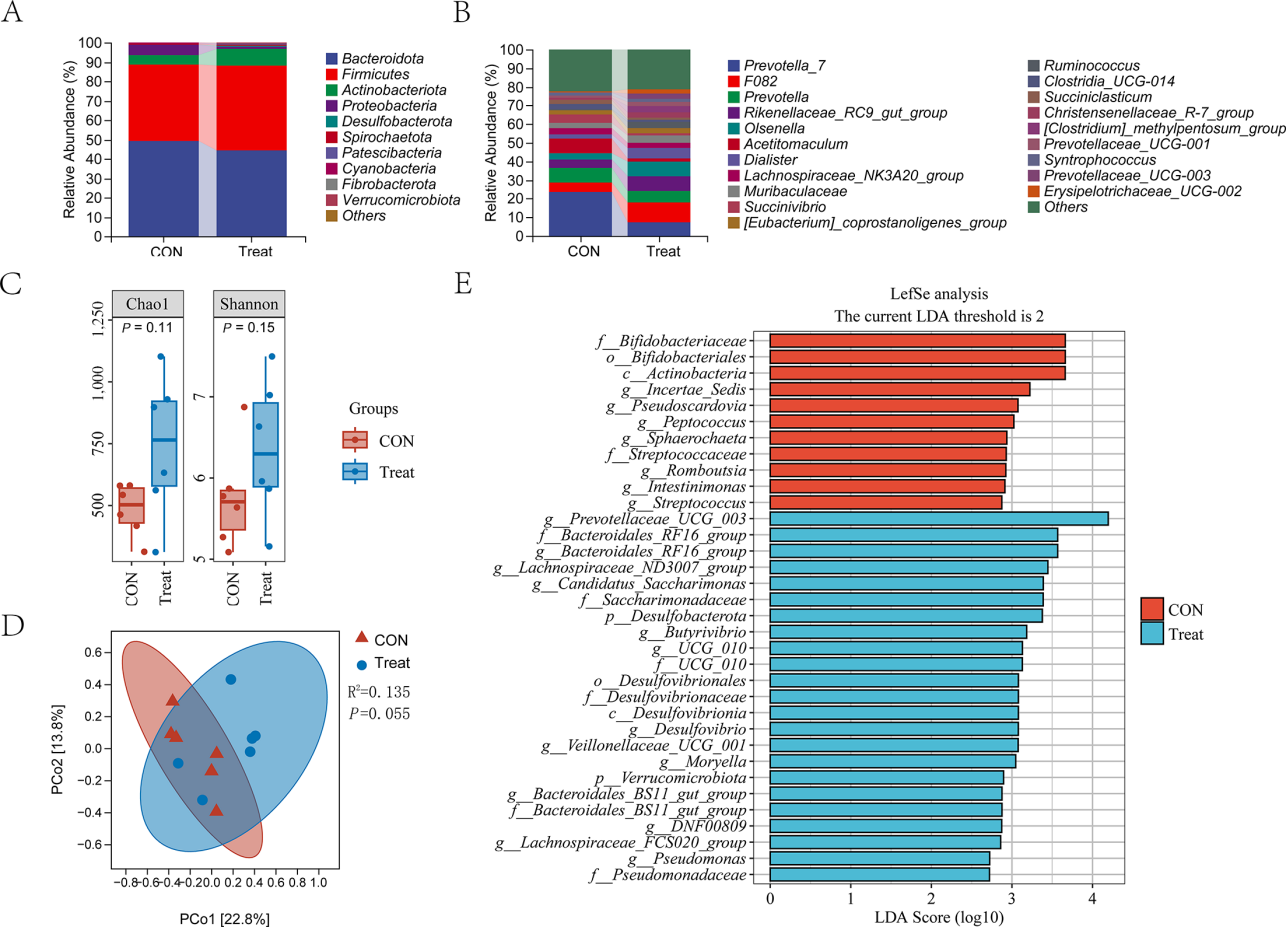
Compositional and differential analysis of ruminal microbiota. (**A**) Phylum-level taxonomic profile. (**B**) Genus-level relative abundance. (**C**) α-Diversity indices. (**D**) Principal coordinate analysis (PCoA) plot based on weighted UniFrac distances. PERMANOVA revealed that FCHM supplementation did not significantly alter hindgut microbial community structure (R² = 0.135, *P* = 0.055). (**E**) Histogram of linear discriminant analysis (LDA) effect sizes across taxonomic ranks; bar length represents LDA score magnitude. Significant discriminative features were defined by *P* < 0.05 and LDA score > 2.

In this study, the dominant phyla within the ruminal microbiota were identified as Bacteroidota, Firmicutes, Actinobacteriota, and Proteobacteria, collectively comprising over 94% of the total relative abundance in both the CON and Treat groups (Fig. 8A). Notably, the Treat group displayed significantly higher relative abundances of Firmicutes and Actinobacteriota compared to the control group. As shown in Fig. 8B, analysis of the top 20 most abundant bacterial genera revealed that *Prevotella*, *F082*, *Rikenellaceae_RC9_gut_group*, *Olsenella*, and *Acetitomaculum* were predominant in both groups. However, the CON group exhibited significantly higher relative abundances of *Prevotella_7*, *Prevotella*, and *Acetitomaculum* compared to the Treat cohort. Furthermore, the relative abundances of *F082*, *Rikenellaceae_RC9_gut_group*, and *Olsenella* were significantly elevated in the Treat cohort compared to CON counterparts (*P* < 0.05), as delineated in Fig. 8B.

α-Diversity analysis showed higher Chao1 richness and Shannon indices in the ruminal microbiota of the Treat group compared to the CON group; however, these differences were not statistically significant (*P* > 0.05), as illustrated by the boxplots in Fig. 8C. PERMANOVA analysis based on weighted UniFrac revealed that dietary FCHM supplementation explained 13.5% of the variation in ruminal microbial community structure, but the difference between groups was not statistically significant (R² = 0.135, *P* = 0.055, Fig. 8D).

LEfSe analysis identified seven microbial genera that were significantly enriched in the ruminal microbiota of lambs in the CON group, with notable genera including *Pseudoscardovia*, *Peptococcus*, *Sphaerochaeta*, and *Streptococcus*. In contrast, 13 genera were significantly more abundant in the Treat group, with key taxa including *Prevotellaceae_UCG-003*, *Bacteroidales_BS11_gut_group*, *Bacteroidales_RF16_group*, *Lachnospiraceae_ND3007_group*, *Candidatus Saccharimonas*, *Butyrivibrio*, and *Desulfovibrio*, among others (Fig. 8E). It is indicated that FCHM supplementation selectively modulated specific bacterial taxa despite minimal changes in overall community diversity and structure.

### Microbial function

**Fig 9:**
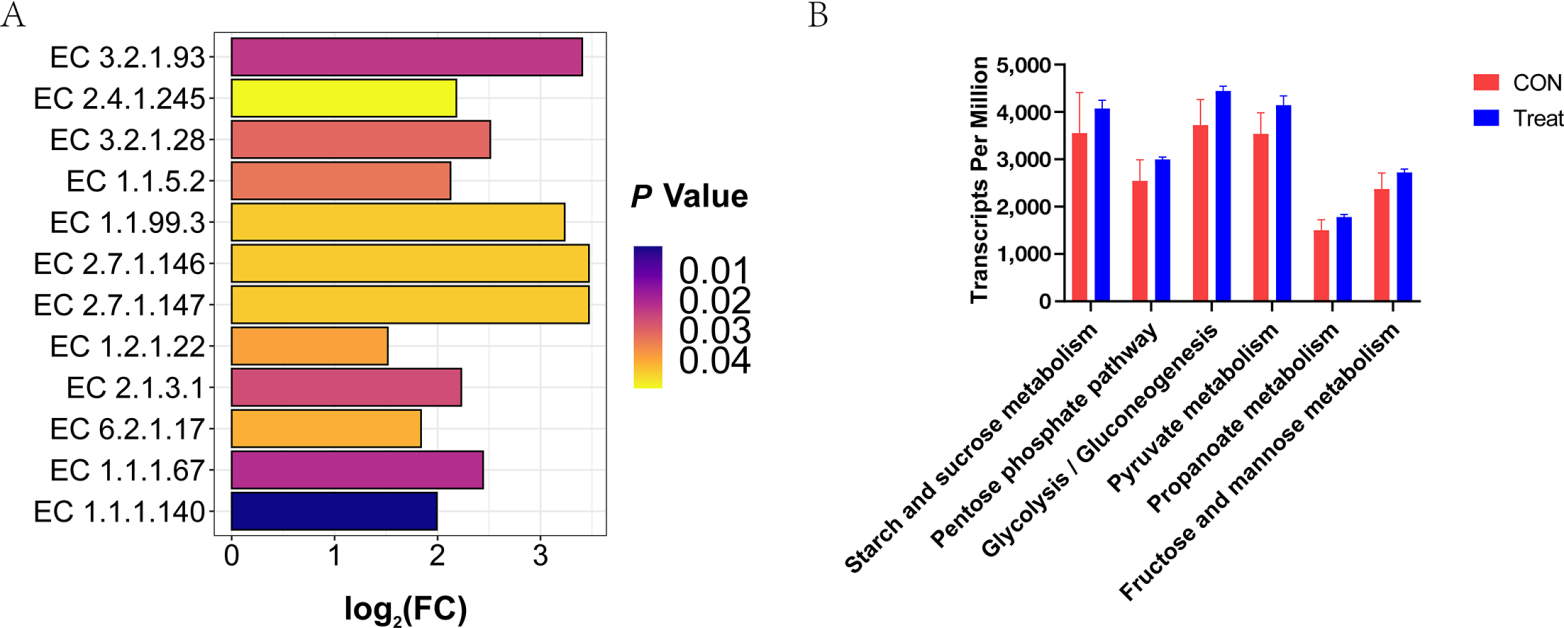
Differential microbial functional profiles in the rumen. (**A**) Differentially abundant enzymes associated with carbohydrate and propionate metabolism derived from bacterial functional prediction, with fold-change (FC) values representing the Treat/CON relative abundance ratio. (**B**) Relative abundance of KEGG related to carbohydrate and propionate metabolism at the Level 3 hierarchy based on metagenomic sequencing.

Functional prediction using PICRUSt2 revealed significant enrichment of 12 KEGG enzymes involved in carbohydrate metabolism and propionate biosynthesis pathways in the Treat group (log₂FC > 1.5; Fig. 9A). In addition, metagenomic sequencing showed markedly higher relative abundances of genes linked to six specific pathways related to carbohydrate utilization and propionate biosynthesis at the KEGG Level 3 hierarchy in the Treat group compared to the CON group (Fig. 9B). Together, these results suggest an enhanced microbial metabolic capacity for carbohydrate breakdown and propionate production in lambs receiving the fermented herbal supplement.

**Fig 10:**
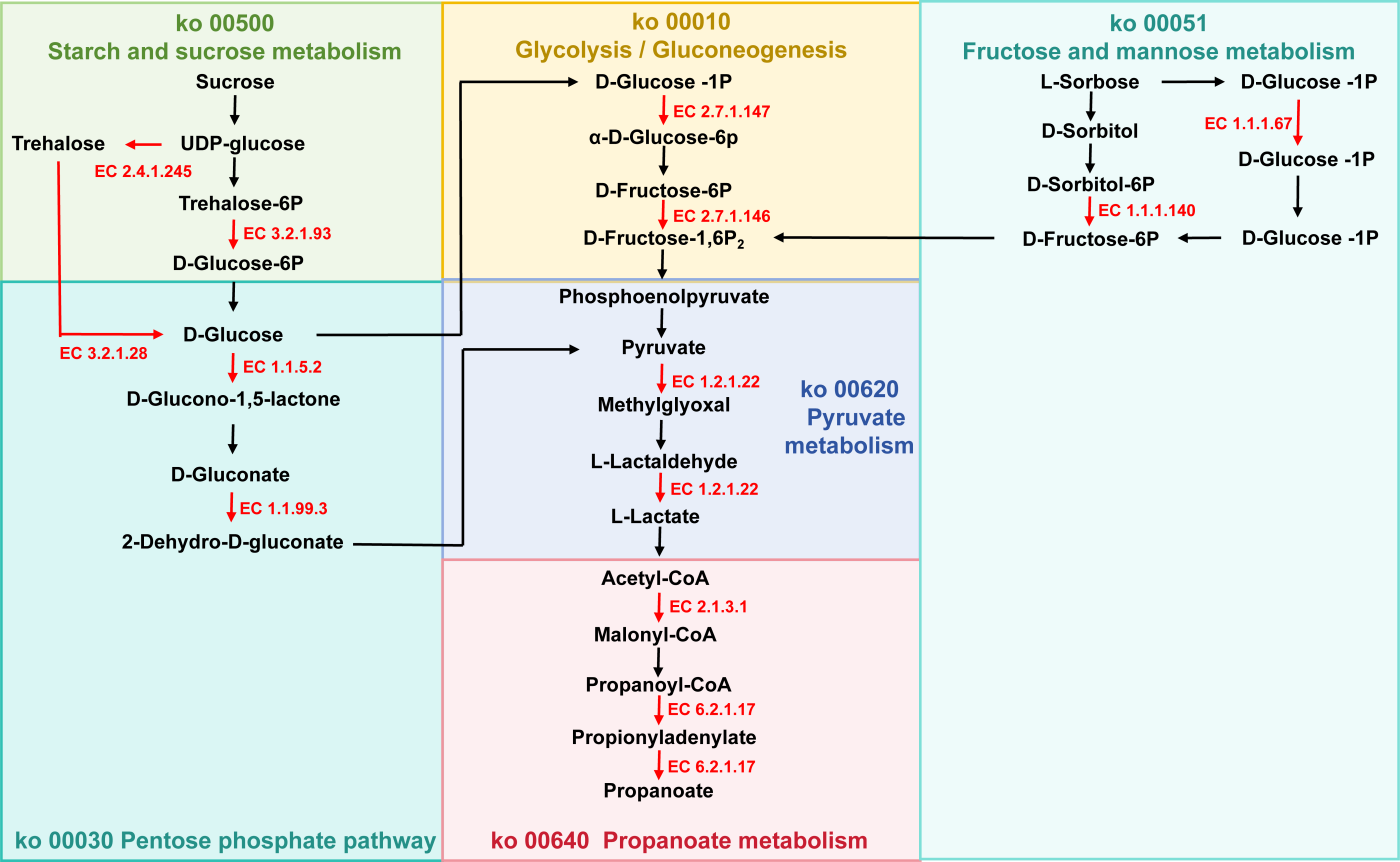
Microbial enzymatic functions associated with carbohydrate and propanoate metabolism in CON versus Treat groups. Red arrows denote enzymes significantly enriched in the Treat cohort (LDA > 2.0, *P* < 0.05), with corresponding annotations indicating catalytic functions.

Figure 10 highlights key KEGG enzymes involved in carbohydrate and propionate metabolism within the ruminal microbiota. Notably, two enzymes (EC 2.4.1.245 and EC 3.2.1.93) in the starch and sucrose metabolism pathway (ko00500) exhibited significantly higher relative abundances in the Treat cohort (log₂FC > 1.5, *P* < 0.05; Fig. 9A). In the glycolytic metabolism and fructose/mannose metabolism pathways, four enzymes were significantly enriched in the Treat cohort: gluconate kinase (EC 2.7.1.147), deoxyglucose kinase (EC 2.7.1.146), D-malate dehydrogenase (EC 1.1.1.140), and mannitol dehydrogenase (EC 1.1.1.67), all demonstrating substantial fold increases (log₂FC > 1.5, *P* < 0.05; Fig. 9A). Additionally, the Treat group showed significantly elevated levels of methylmalonate-semialdehyde dehydrogenase (EC 1.2.1.22), a key enzyme in the pyruvate metabolism pathway. In the pentose phosphate pathway (ko00030), three enzymes, namely gluconate 2-dehydrogenase (EC 1.1.5.2), glucose dehydrogenase (EC 1.1.99.3), and endo-1,3-β-glucosidase (EC 3.2.1.28), were significantly enriched in the Treat group. Concurrently, enzymes linked to propionate biosynthesis, methylmalonyl-CoA carboxyltransferase (EC 2.1.3.1) and succinate-CoA ligase (EC 6.2.1.17), demonstrated significantly higher abundances in the treatment group relative to CON (log₂FC > 1.5, *P* < 0.05; Fig. 9A).

### Differential carbohydrate-active enzymes

**Fig 11:**
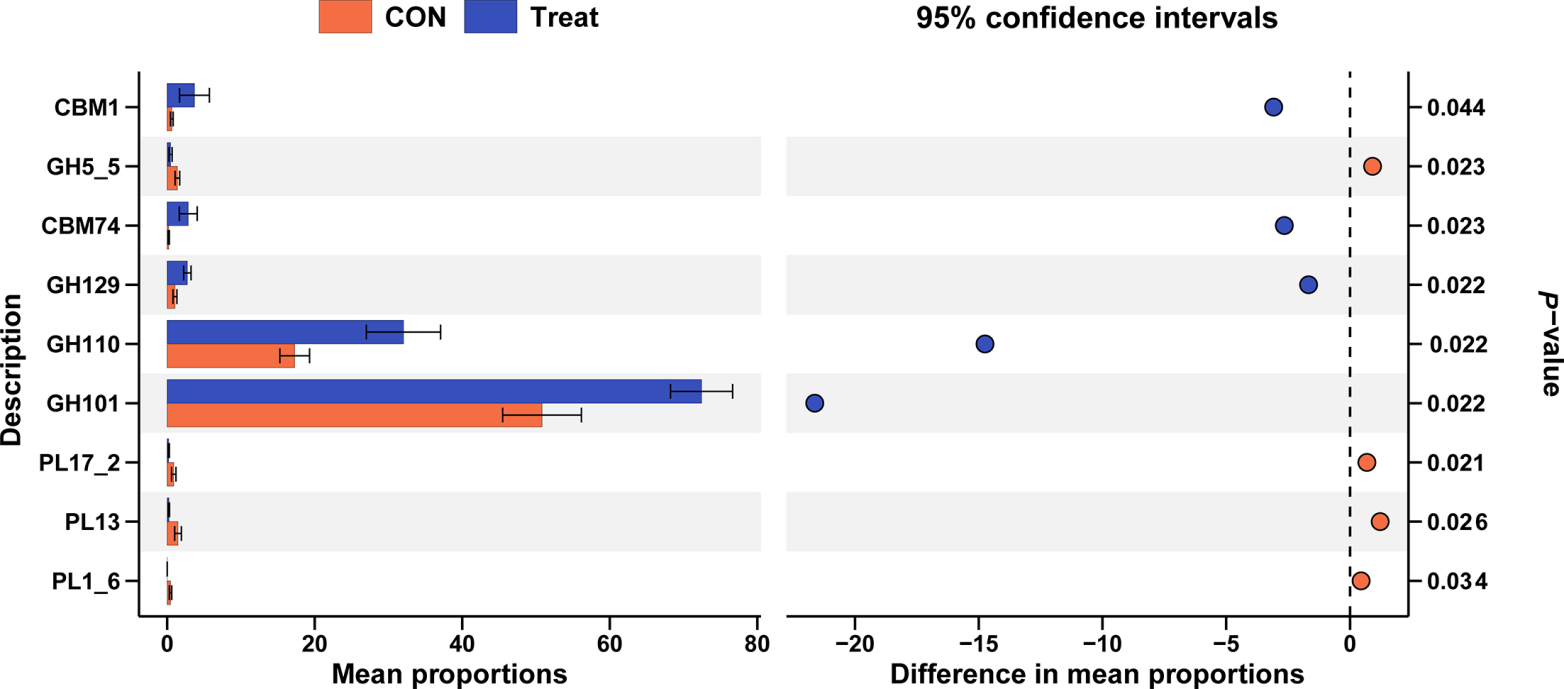
Differential CAZyme family profiles in ruminal metagenomes between experimental groups.

Metagenomic analysis revealed nine differentially abundant CAZyme genes within the ruminal microbiome (Fig. 11), including two carbohydrate-binding module (CBM) genes, four glycoside hydrolase (GH) genes, and three polysaccharide lyase (PL) genes. In the Treat group, significant enrichment was observed in CAZyme families CBM1, CBM74, GH129, GH110, and GH101. In contrast, the CON group showed significantly higher abundances of GH5_5, PL17_2, PL13, and PL1_6.

### Correlation analysis

**Fig 12:**
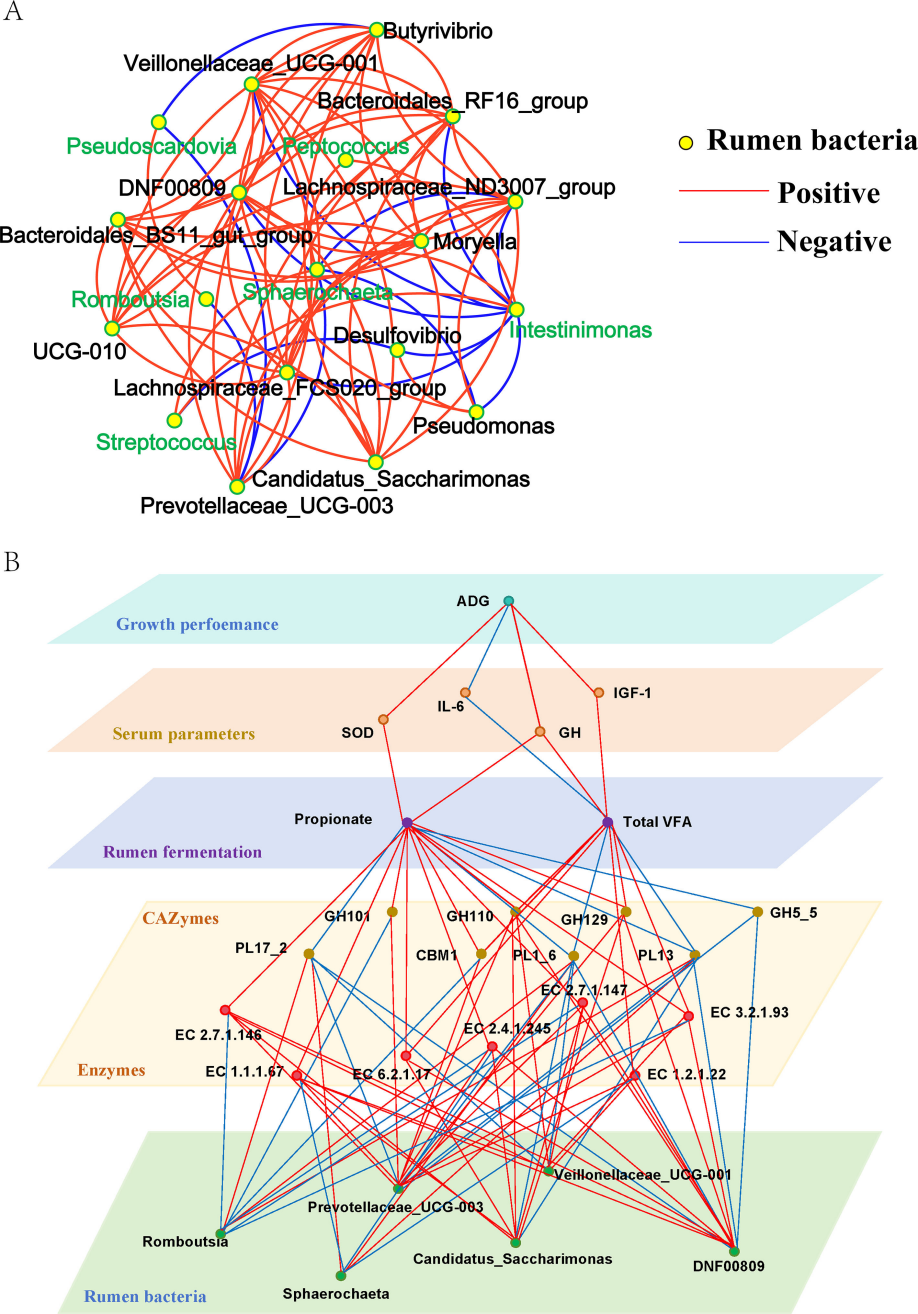
Spearman correlation networks. (**A**) Interrelationships among differentially abundant bacterial genera; green labels denote taxa significantly enriched in CON, and black labels indicate enrichment in Treat. (**B**) Multiplex network analysis revealed the interrelationships among ruminal bacteria, KEGG enzymes, CAZymes, rumen fermentation parameters, serum indices, and host phenotypes. The edges between nodes represent correlations, with red and blue edges indicating significant positive and negative correlations, respectively (Spearman’s |r| > 0.50, *P* < 0.05).

In this study, Spearman correlation network analysis revealed intricate interactions among the ruminal microbiota (Fig. 12A). Genera significantly enriched in the Treat group, such as *Bacteroidales_BS11_gut_group*, *Butyrivibrio*, *Lachnospiraceae*, and *Prevotellaceae_UCG-003,* exhibited notably higher network connectivity, suggesting enhanced metabolic interactions and positioning them as key hubs within the microbial community. In contrast, genera enriched in the CON group, such as *Peptococcus*, *Pseudoscardovia*, *Romboutsia*, *Moryella*, *Sphaerochaeta*, and *Streptococcus,* exhibited lower network connectivity, indicating a reduced topological role within the overall microbial interaction network.

We further constructed a multi-network to visualize Spearman rank correlation coefficients, thereby investigating the interrelationships among growth performance, serum parameters, rumen fermentation, enzymes, CAZymes, and rumen bacteria (Fig. 12B). Specifically, the relative abundance of *Prevotellaceae_UCG-003* exhibited significant positive correlations with the enzymes EC 3.2.1.93, EC 2.4.1.245, EC 1.1.5.2, EC 1.1.99.3, EC 2.7.1.146, EC 2.7.1.147, and EC 1.1.1.67. Furthermore, it was also positively correlated with the CAZymes GH101, GH110, GH129, and CBM1 (*P* < 0.05). The relative abundance of *Veillonellaceae_UCG-001* exhibited significant positive correlations with the enzymes EC 3.2.1.93, EC 3.2.1.28, EC 1.1.1.67, EC 1.2.1.22, EC 2.7.1.147, and EC 2.7.1.146, as well as with the CAZyme GH110 (*P* < 0.05).

Both *Candidatus_Saccharimonas* and *DNF00809* exhibited significant positive correlations with the enzymes EC 2.4.1.245, EC 3.2.1.28, EC 2.7.1.146, EC 2.7.1.147, EC 6.2.1.17, and EC 1.1.1.67, as well as with the CAZymes GH110 and GH129 (*P* < 0.05). A significant positive correlation was observed between propionate and several parameters, including EC 3.2.1.93, EC 2.7.1.146, GH 101, as well as serum SOD and GH (*P* < 0.05). Concurrently, total VFA levels were positively correlated with EC 3.2.1.93, EC 6.2.1.17, GH110, and the serum hormones GH and IGF-1 but negatively correlated with IL-6 (*P* < 0.05). Furthermore, serum SOD, GH, and IGF-1 demonstrated positive correlations with ADG (*P* < 0.05).

### Hindgut microbiota composition, diversity, and differential abundance

**Fig 13:**
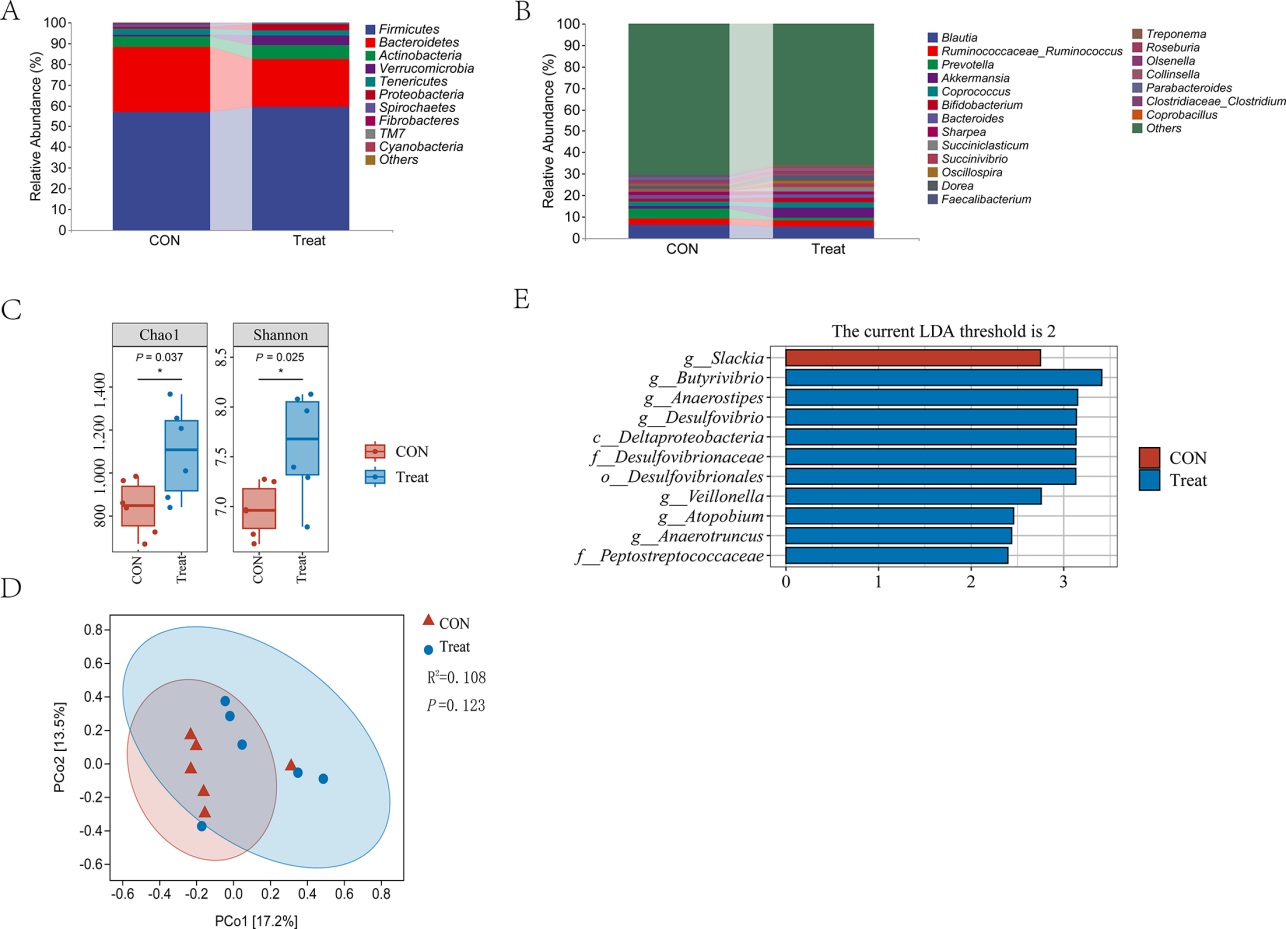
Fecal bacterial composition and differential features. (**A**) Phylum-level taxonomy. (**B**) Genus-level taxonomy. (**C**) α-Diversity indices. (**D**) Principal coordinate analysis (PCoA) plot based on weighted UniFrac distances. PERMANOVA revealed that FCHM supplementation did not significantly alter hindgut microbial community structure (R² = 0.108, *P* = 0.123). (**E**) Histogram of linear discriminant analysis (LDA) scores across bacterial taxa, with bar length representing LDA effect size. Significant discriminative features were defined as LDA score > 2 and *P* < 0.05.

Analysis of the effects of fermented Chinese herbal compounds on hindgut microbial composition revealed that Bacteroidota, Firmicutes, Bacteroidetes, and Actinobacteria formed the core microbiota, collectively accounting for over 89% of the total relative abundance in both the CON and Treat groups (Fig. 13A). Notably, the Treat group showed significantly higher relative abundances of Firmicutes and Actinobacteriota compared to the control group. At the genus level, the Treat group exhibited substantial enrichment of *Blautia*, *Ruminococcaceae_Ruminococcus*, *Prevotella, Akkermansia*, *Bifidobacterium*, and *Succiniclasticum*, which collectively represented the dominant taxa in the fecal microbiota (Fig. 13B). As depicted in Fig. 13C, the Treat group also demonstrated significantly higher Chao1 richness and Shannon diversity indices, indicating enhanced microbial richness following dietary supplementation with the fermented herbal compound. PCoA further revealed a partial separation between the CON and Treat groups. PERMANOVA, based on weighted UniFrac distances, revealed that FCHM supplementation explained 10.8% of the variation in hindgut microbial community structure, but the difference was not statistically significant (R² = 0.108, *P* = 0.123; Fig. 13D). LEfSe analysis identified one distinct biomarker genus (*Slackia*) in the CON group, while the Treat group exhibited six significantly enriched genera, including *Butyrivibrio*, *Anaerostipes*, *Desulfovibrio*, *Veillonella*, *Atopobium*, and *Anaerotruncus* (Fig. 13E).

## DISCUSSION

The heightened global concern for animal health and food safety has stimulated rigorous exploration of effective antibiotic alternatives in livestock production. Among these, herbal compounds utilized as feed additives have attracted considerable scientific interest. Accumulating evidence underscores their potential as antimicrobial substitutes, thereby establishing plant-derived supplementation as a pivotal field of investigative focus (14). This study demonstrates that dietary supplementation with fermented compound Chinese herbal medicine during the lamb suckling period significantly enhances growth performance, rumen fermentation, and antioxidant capacity. These improvements are closely associated with a restructuring of the gastrointestinal microbiota and enhanced metabolic pathways (15). Characterization of the fermented herbal formulation identified alkaloids and flavonoids as the principal bioactive constituents, with the major phytochemicals comprising betaine, tangeretin, hesperetin, naringenin, and hesperidin. In ruminants, dietary intake of flavonoids appears to modulate the ruminal microbial community, thereby influencing host growth dynamics and nutrient absorption processes (16). Substantial evidence from previous investigations corroborates the beneficial effects of various flavonoids on lamb growth, carcass characteristics, and meat quality. For instance, Ghamdi et al. (17) documented that dietary supplementation with citrus flavonoids improved ADG, ruminal papilla development, carcass weight, and meat quality parameters. These findings suggest that citrus flavonoids promote rumen maturation and enhance nutrient absorption, thereby contributing to increased body weight. Similarly, Hao et al. (18) reported that moderate supplementation of sea buckthorn flavonoids improved nutrient digestibility, optimized rumen fermentation, reduced urinary nitrogen excretion, and enhanced antioxidant status and growth performance in lambs—findings consistent with our observations. In the present study, no significant difference was observed in starter diet intake between the two lamb groups. However, lambs receiving the diet containing 0.6% fermented compound herbs exhibited a significantly higher ADG compared to the control group (Fig. 2). This suggests that the observed growth-promoting effects were primarily driven by enhanced feed utilization efficiency and improved nutrient absorption rather than increased feed intake. These benefits are likely mediated by bioactive constituents present in the fermented herbal additive, which support metabolic regulation, reinforce immune responses, and mitigate oxidative stress and inflammation, collectively contributing to physiological conditions favorable for improved ADG.

Further analysis of serum parameters (Fig. 3) revealed that lambs fed the fermented herbal compound exhibited significantly elevated levels of GLU, GH, IGF-1, GSH-Px, T-AOC, and SOD compared to the control group. As a fundamental energy-yielding nutrient in mammals, GLU is critically associated with energy intake and growth performance (19). In ruminants, glycemic homeostasis demonstrates a positive correlation with glucose turnover, suggesting that elevated circulating glucose levels promote glucose metabolism. GH and IGF-1 orchestrate growth and development through coordinated hepatic metabolism. Previous investigations have demonstrated that dietary betaine supplementation alleviates stress responses and elevates both plasma concentrations and mRNA expression levels of GH and IGF-1 in heat-stressed dairy cows, consequently improving feed intake and enhancing growth performance (20). Furthermore, research indicates that dietary supplementation with citrus flavonoids significantly increases ruminal propionate concentration in dairy cows (21), which aligns with our current findings and suggests an enhanced availability of gluconeogenic precursors. The concurrent elevation of serum glucose and growth-related hormones indicates a coordinated enhancement of glucose metabolism, likely driven by increased availability of energetic substrates. This metabolic shift may activate physiological pathways associated with growth regulation, thereby supporting improved developmental outcomes in lambs. These findings align with previous observations reported by Xu in calves (22). Adequate energy supply is essential for both the synthesis of endogenous antioxidants and the maintenance of cellular redox homeostasis. In the present study, the positive correlations observed among propionate, total VFA, and serum levels of SOD and GH further support this energy-mediated upregulation of the antioxidant system (Fig. 12B).

The rumen plays a crucial role in nutrient digestion and absorption in ruminants, with its fermentation parameters serving as key indicators of fermentation efficiency and the overall ruminal microenvironment. Propionate, valerate, and isobutyrate function as precursors for hepatic gluconeogenesis, representing primary sources of glucose and energy in ruminants. Notably, propionate contributes to 60%–74% of hepatic glucose production (23). In the present study, dietary supplementation with the fermented herbal compound significantly increased the concentrations of propionate and TVFA in the rumen of lambs (Fig. 7). This improvement may be attributed to the proliferation of functional microbial populations and enhanced ruminal enzymatic activity, thereby elevating energy availability and supporting growth development. These findings align with observations by Wang et al., who reported elevated propionate levels with a decreased acetate-to-propionate ratio during *in vitro* fermentation of Pogostemon cablin, Smilax china, and Phellodendron chinense (24). Collectively, these results indicate a shift in ruminal fermentation patterns toward enhanced energy utilization efficiency, thereby supporting the improved growth performance observed in lambs. Similarly, supplementation with Yupingfeng San has been shown to increase the concentrations of propionate while promoting growth performance, which aligns with our observations (25). Collectively, the evidence indicates that the fermented compound herbal additive contributes to an improved ruminal microenvironment. The rumen microbiota plays a pivotal role in ruminant productivity, supplying approximately 70% of the host’s energy requirements through VFA metabolism (26). In the present study, 16S rRNA high-throughput sequencing analysis revealed that the ruminal bacteria of suckling lambs supplemented with the fermented herbal compound did not show significant differences in either the Chao1 index or the Shannon diversity index (Fig. 8C and D). This indicates that supplementation induced a targeted restructuring of the ruminal bacterial community without eliciting broad changes in microbial richness or diversity. Previous studies have demonstrated that interactions between flavonoids and gut microbiota are key to mediating the health benefits of plant-derived flavonoids in non-ruminant species (27), suggesting a potentially analogous mechanism in ruminants. Citrus flavonoids modulate gastrointestinal microbial composition by promoting the proliferation of beneficial bacteria while suppressing pathogenic species. Concurrently, the gut microbiota enhances the bioavailability of flavonoids and transforms them into bioactive metabolites (28). These reciprocal interactions likely contribute to the mitigation of oxidative stress and inflammatory responses within the rumen, thereby fostering microbial growth and improving nutrient absorption. Furthermore, emerging evidence suggests that plant-derived flavonoids may serve as carbon sources for ruminal microorganisms, which could explain the observed increase in total VFA concentrations (29).

The FCHM itself provides direct antioxidant constituents, with flavonoids such as hesperetin and naringenin serving as potent free radical scavengers and metal chelators. Emerging evidence indicates that citrus flavanones modulate gut microbial composition and activity by inhibiting pathogenic bacteria while selectively promoting the growth of beneficial microorganisms (30). In our study, we identified 13 bacterial phyla within the rumen (Fig. 8E). Notably, the abundance of *Prevotellaceae_UCG-003* involved in carbohydrate and protein metabolism was significantly increased following supplementation with fermented herbs (9). This microbial shift aligns with our findings and may thereby contribute to the elevated ruminal propionate levels observed in supplemented lambs. Supporting this observation, Wang et al. also reported a positive correlation between the abundance of *Prevotellaceae_UCG-003* and propionate concentration in the rumen (24). Furthermore, *Veillonellaceae_UCG-001*, known for its role in cellulose degradation and utilization, exhibited a similar increasing trend. Previous studies have demonstrated that supplementation with probiotics and herbal polysaccharides modulates rumen fermentation patterns, increases the abundance of *Veillonellaceae_UCG-001*, and improves growth performance in lambs compared to controls (31). These observations are highly consistent with our current data. Meanwhile, *Bacteroidales BS11_gut_group* and *Bacteroidales RF16_group* contribute to host energy metabolism by producing acetate via the pyruvate-acetyl-CoA pathway and propionate through the succinate pathway, respectively. Dietary supplementation with citrus peel extract has been shown to enhance the abundance of *Bacteroidales RF16_group* in the rumen of transition dairy cows (32). These findings suggest that flavonoids, such as hesperidin, modulate the ruminal microbial community in a manner that supports enhanced host energy metabolism. Phylogenetically classified within the *Ruminococcaceae* family, *UCG-001* belongs to a bacterial group recognized for its importance in plant fiber degradation (33). Concurrently, *Butyrivibrio* plays a central role in ruminal butyrate production, exerts anti-inflammatory properties, and upregulates the expression of antioxidant enzymes such as GSH-Px and SOD in intestinal epithelial cells, potentially eliciting systemic effects. Although ruminal butyrate levels did not increase significantly, the enrichment of butyrate-producing genera underscores its potential functional relevance to the SCFA pool and intestinal health. Numerous bacterial strains within the *Lachnospiraceae* family are also involved in the metabolism of flavanones, including hesperidin and naringin (34). Our findings corroborate these observations, reinforcing their validity. *Moryella,* another genus within the *Lachnospiraceae* family, is known to produce carbohydrate-degrading enzymes and generate volatile fatty acids (35). *Pseudomonas* species also contribute significantly by secreting lignocellulose-degrading enzymes such as GH 11 and GH 43, which facilitate fiber breakdown and contribute to the reduction of methane emissions (36). Research has established *Desulfovibrio* as a sulfate-reducing bacterium in the rumen of ruminants, capable of producing sulfide that inhibits short-chain fatty acid (SCFA) oxidation (37). In our study, an increased abundance of *Desulfovibrio* was observed without apparent adverse effects, which may be attributable to differences in dietary composition or animal genetic background. However, the underlying mechanisms require further investigation. Interestingly, genera such as *Peptococcus*, *Pseudoscardovia*, *Sphaerochaeta*, and *Streptococcus* were more abundant in the CON group but showed reduced relative abundance in the treatment group. In addition, *Peptococcus* may contribute to ruminal acidosis (38). Furthermore, elevated abundance of *Pseudoscardovia* in early-weaned lambs has been associated with reduced microbial diversity and may impede the reconstruction of beneficial microbiota (39). In our study, the significant reduction of *Pseudoscardovia* in the treatment group likely resulted from microbial interactions, as supported by bacterial correlation analysis (Fig. 12A). *Sphaerochaeta*, linked to reduced fat deposition in swine and poultry, could potentially interfere with lipid metabolism during rapid growth phases in lambs if overproliferated, thereby adversely affecting growth performance (40). *Streptococcus*, a well-established contributor to ruminal acidosis, can inhibit the activity of fibrolytic bacteria (41). Collectively, supplementation with the fermented herbal compound effectively reduced the abundance of detrimental rumen bacteria, thereby fostering a more favorable microbial environment conducive to optimal growth and development in lambs.

Rumen bacteria play a central role in lignocellulose decomposition and VFA production through the secretion of CAZymes, which convert complex plant fibers into soluble sugars for subsequent fermentation into VFAs (42). In the present study, five CAZyme families were found to be enriched in the treatment group (Fig. 11). Among these, GH101 specifically catalyzes the targeted hydrolysis of mucins to release readily bioavailable carbon sources (43). CBM1 enhances enzyme-substrate binding to crystalline cellulose, thereby improving fiber hydrolysis efficiency, while CBM74 selectively binds granular starch and facilitates the degradation of resistant starch through synergistic action with α-amylase (44). Further functional prediction using PICRUSt2 revealed the enrichment of 12 KEGG enzymes associated with carbohydrate and propionate metabolism in the treatment group (Fig. 10). Notably, ADP-dependent phosphofructokinase (EC 2.7.1.146) catalyzes the conversion of fructose-6-phosphate to fructose-1,6-bisphosphate, while ADP-dependent glucokinase (EC 2.7.1.147) phosphorylates glucose to form glucose-6-phosphate. These enzymes collectively enhance glycolytic flux, facilitating the generation of pyruvate precursors essential for energy metabolism (45). In addition, sorbitol-6-phosphate dehydrogenase (EC 1.1.1.140) promotes the reversible interconversion between fructose-6-phosphate and sorbitol-6-phosphate, and mannitol dehydrogenase (EC 1.1.1.67) participates in the mutual conversion of fructose and mannitol. These enzymatic activities help redirect carbon flow through the fructose and mannose metabolic pathways, thereby supporting the synthesis of pyruvate precursors (46). Lactaldehyde dehydrogenase (EC 1.2.1.22) catalyzes the conversion of lactaldehyde to pyruvate. Concurrently, transcarboxylase (EC 2.1.3.1) mediates the transfer of a carboxyl group from methylmalonyl-CoA to pyruvate, yielding propionyl-CoA and oxaloacetate—key intermediates in propionate metabolism (47, 48). Propionyl-CoA synthetase (EC 6.2.1.17) activates propionate to propionyl-CoA and also contributes to propionate biosynthesis (49). Furthermore, D-gluconate dehydrogenase (EC 1.1.99.3) and glucose dehydrogenase (EC 1.1.5.2) oxidize glucose to gluconate, which subsequently enters the pentose phosphate pathway, thereby indirectly supporting energy metabolism (50, 51). The collective upregulation of these KEGG enzymes indicates enhanced carbohydrate catabolism through multiple pathways, including fructose and mannose metabolism, starch and sucrose metabolism, pentose phosphate pathway, and glycolysis. Simultaneously, these enzymatic activities support propionate metabolism via pyruvate processing, contributing to increased ruminal propionate production. Microbial interaction network analysis revealed that the relative abundance of *Prevotellaceae_UCG-003* showed significant positive correlations with multiple KEGG enzymes involved in starch/sucrose metabolism and the pentose phosphate pathway, as well as various glycoside hydrolases (Fig. 12B). This suggests that this microbial genus serves as a key executor in mediating FCHM-enhanced ruminal carbohydrate degradation capacity. Similarly, *Veillonellaceae_UCG-001* and Candidatus_Saccharimonas were positively correlated with key enzymes in glycolytic and propionate-generating pathways. Propionate concentration demonstrated positive correlations with starch-degrading enzyme (EC 3.2.1.93), glucokinase (EC 2.7.1.146), and serum levels of SOD and GH. These findings indicate that specific microbial taxa enriched by FCHM directly drive the activation of carbohydrate catabolism and propionate generation pathways in the rumen, thereby enhancing systemic energy availability to support lamb growth. Furthermore, the beneficial microbial community appears to more effectively release and metabolize bioactive compounds from the herbal additive, which in turn further supports microbial homeostasis and reduces ruminal oxidative stress. The subsequent decrease in serum pro-inflammatory cytokines (IL-6 and TNF-α) likely reflects this improvement in ruminal and systemic antioxidant status, given the established interconnection between oxidative stress and inflammation. These correlative relationships substantially strengthen the mechanistic explanation for FCHM’s mode of action.

Accumulating evidence underscores the significant contribution of the hindgut microbiota to ruminant health and production efficiency (52). Notably, under high-grain dietary conditions, nearly 50% of dietary starch escapes ruminal digestion and enters the lower gastrointestinal tract, where it undergoes further processing by intestinal microorganisms (53). Recent investigations have highlighted the crucial role of gut microbiota in transforming citrus flavonoids into bioactive metabolites (54). While ruminal bacteria partially deglycosylate compounds such as naringin and hesperidin (55), unmetabolized flavonoids typically progress to the hindgut alongside undigested nutrients. There, accumulated flavonoid aglycones are further catabolized by gut microorganisms into bioactive derivatives. Emerging studies in non-ruminant species demonstrate that gut microbiota-mediated metabolism can activate or potentiate the inherent bioactivity of citrus flavonoids, thereby generating beneficial effects on host health (56). In the present study, analysis of the fecal microbiota revealed a significantly higher alpha diversity in lambs supplemented with the fermented herbal compound, indicating a substantial influence on the hindgut microbial community. This increase was driven by enhanced richness and evenness (Fig. 13C), with enrichment of specific taxa such as *Butyrivibrio* and *Anaerostipes* (Fig. 13E), likely attributable to undigested herbal components exerting prebiotic effects or reducing competitive exclusion in the hindgut. Previous research has demonstrated that citrus flavonoids inhibit the growth and adhesion of pathogenic bacteria to human intestinal cells, while simultaneously enhancing the growth and adhesion of probiotic strains (57). Supplementation studies in dairy cows have shown that citrus flavonoids increase the relative abundance of beneficial genera, such as *Bifidobacterium* and *Akkermansia*, in the hindgut (58). Our findings in lambs similarly revealed an enrichment of *Butyrivibrio*, *Desulfovibrio*, *Veillonella*, *Atopobium*, and *Anaerotruncus* in the treatment group. Notably, *Butyrivibrio* plays a crucial role in decomposing non-starch polysaccharides into SCFAs, which contribute to the maintenance of intestinal barrier function and immune homeostasis. Furthermore, *Butyrivibrio* may influence host immunometabolic responses by modulating gut microbial composition (59). Similarly, *Veillonella* ferments lactate to produce SCFAs that are essential for regulating immune function, energy metabolism, and maintaining intestinal homeostasis (60). *Atopobium*, an anaerobic genus within the *Actinobacteria* phylum, is commonly found in mammalian gastrointestinal tracts. However, its specific functional role in the ruminant gut remains incompletely characterized and warrants further investigation. In contrast, *Anaerotruncus* is closely associated with intestinal barrier integrity and inflammatory regulation. This genus participates in carbohydrate fermentation to produce butyrate, which is critical for maintaining intestinal mucosal integrity. Moreover, butyrate may function as a signaling molecule that upregulates antioxidant enzymes such as SOD and GSH-Px in distal organs through mechanisms including histone deacetylase inhibition or G-protein-coupled receptor activation. Therefore, hindgut-derived butyrate may serve as an important signaling mediator indirectly participating in the activation of antioxidant programs in the duodenal mucosa (61). Collectively, supplementation with the fermented compound herbal additive appears to promote the colonization of beneficial microorganisms in the hindgut. This likely enhances nutrient absorption efficiency through butyrate-mediated support of intestinal barrier function while reducing systemic inflammatory and oxidative burdens, thereby contributing to improved growth performance in lambs. Although alterations in gut microbiota and serum biomarkers have been documented, direct histopathological examinations of intestinal mucosa, villus architecture, or ruminal papillae were not conducted. Consequently, subsequent studies should prioritize direct validation of these findings through integrated histopathological, molecular, and immunological assessments, complemented by targeted metabolomic analyses to elucidate the underlying metabolic mechanisms. Simultaneously, the fermentation process warrants further optimization. Although the current protocol effectively enhanced lamb performance, further optimization of key parameters using response surface methodology or dynamic metabolite monitoring could maximize the bioactivity and consistency of fermented herbal products.

### Conclusions

**Fig 14:**
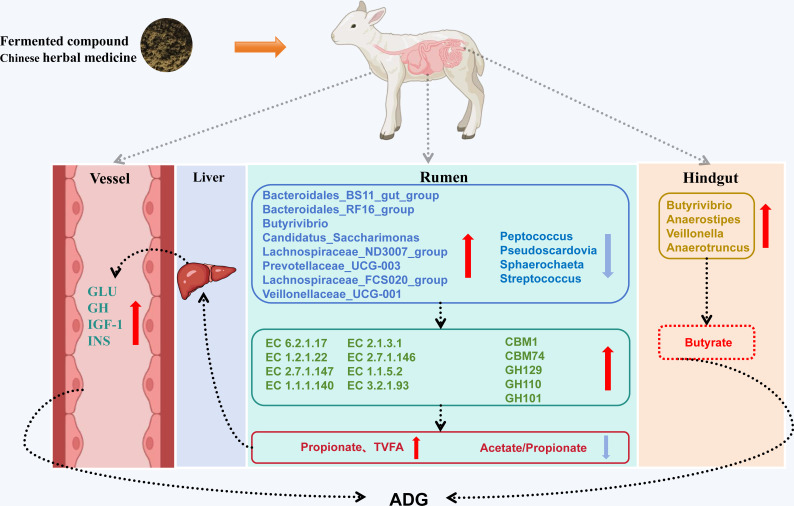
Fermented compound Chinese herbal medicine drives lamb growth by reshaping the gastrointestinal microbiota and enhancing antioxidant capacity.

This study demonstrates that dietary supplementation with FCHM significantly enhanced lamb growth performance, as evidenced by increased average daily gain. Furthermore, FCHM restructured the ruminal microbiota by enriching beneficial taxa and suppressing harmful bacteria, thereby optimizing fermentation patterns particularly through elevated propionate and total volatile fatty acid concentrations, which consequently improved host energy status. Concurrently, the direct antioxidant effects of bioactive constituents, combined with anti-inflammatory outcomes derived from specific microbial metabolites, synergistically enhanced systemic antioxidant capacity. The resulting mitigation of oxidative stress and inflammation established a physiological milieu conducive to efficient nutrient utilization and robust growth (Fig. 14). These findings support FCHM as a promising antibiotic-alternative feed additive for improving lamb health and productivity, although further studies including histopathological validation are warranted.

## Data Availability

The metabolomic profiling data are available in the MetaboLights repository and can be accessed via the following: https://www.ebi.ac.uk/metabolights/editor/MTBLS12888/descriptors. The 16S rRNA gene sequencing and metagenomic sequencing data are available in the NCBI Sequence Read Archive (PRJNA1276716 and PRJNA1276290).
